# The Use of Silver Foil in Dentistry

**Published:** 1901-12-15

**Authors:** L. S. Chilcott

**Affiliations:** Bangor, Me.


### THE USE OF SILVER FOIL IN DENTISTRY.

BY L. S. CHILCOTT, D.D.S., BANGOR, ME.

Read before the American Academy of Dental Science, Boston, May 1, 1901.

On this necessarily brief subject—the use of silver foil in dentistry—I can not hope to offer anything entirely new, but may possibly suggest a new application of that which is very old.

Silver has, in all probability, not changed since it was originally deposited, and physiological conditions have changed very little since the deterioration of the human teeth made dentistry a necessity. It is our knowledge of sepsis, acquired within the last few years, that causes us to be constantly reaching for something that will arrest the growth of bacteria and render our operations permanently aseptic.

The therapeutical value of silver in surgery was recognized ages before the germ theory was ever thought of. Silver plate was formerly used in trephining operations; silver wire is quite generally used for sutures at the present time, and silver foil has been used, I believe, for about two or three years in permanent dressings for wounds that are considered free from infection.

I am unable to find any reference to silver in the form of a foil in dentistry except in the old text-books, where it is mentioned as being too susceptible to oxidation and as lacking sufficient pliability to permit its use as a filling material.

It was my good fortune last summer to have for a patient a very bright and intelligent young lady who is a senior in the medical department of Johns Hopkins University, and she inquired why I never used silver foil. I told her that it was too harsh a material for me to do anything with. "But I beg your pardon," she replied; "it is so soft and loose in its texture as to make it difficult to handle, and really I do not see why it would not be useful to you, and when I return I will send you some of it." It came in due time, and I must admit that I found it as represented, not omitting the difficulty in its manipulation. I usually take about ten sheets and work it into a rope as best I can. It will not remain in form like gold foil, but has a loose, fluffy appearance. I have some of it here, and you will see that it is very thin and requires much care in handling. It is not even suggested that it, by itself, is a practical filling material, but it has its use, however, and I have had such good results that I should not want to be without it.

Silver in its solid form ranks high as a conductor of heat. But this material in the form of a foil, when placed in the cavity of a tooth, appears to be a very efficient barrier against thermal changes. This is attributed to the innumerable interstices and oxidation, and the chemist may determine just what structural changes occur.

Its use in cavities of decay in the oral teeth is not recommended, as it is liable to stain the dentin, but it will in many cases commend itself as an adjunct filling material in the posterior teeth. Theoretically it is all wrong to use this foil in connection with amalgam, as it has a strong affinity for mercury, but it appears to work all right in practice, so far as I have been able to determine from the few cases in which I have used it, and time alone will determine the value of this combination.

In that large class of cases where a non-conductor and eburnification is desired, I can conceive of no better substance for a tooth preservative than this silver foil in connection with that good, old-fashioned, non-cohesive gold foil. With patients where this treatment would be advantageous, and there are thousands of them between the ages of eight and twenty—those, in fact of all ages whose teeth are of faulty development—and in senile decay the cavity should be prepared exactly as a conservative dentist would prepare one for any metallic filling, and the bottom of the cavity given what would be considered a good, thick covering of the silver foil, allowing it to extend up to the enamel in a thin layer on all sides, and the remaining portion of the cavity filled with such material as the judgment of the operator may dictate.

Another use in which I have found this material very desirable is in the filling of pulp canals. It, like everything else that I have ever used for this purpose, is not easily carried into small, inaccessible roots, but with care and the use of smooth instruments it can be put into nearly every pulp canal that any one would attempt to fill, without causing any irritation whatever. I have been surprised and delighted with the results from its use in pulp canals in roots which have a large apical foramen or a perforation. I invariably place a little of it at the apex of pulp canals before setting crowns. In one case, where there was a good-sized opening through the side of a central incisor root which I wished to crown, I stopped the perforation carefully with this foil, secured the crown in position with cement, and the tooth has given no trouble since. I have in many cases in the last few months opened into teeth with putrescent pulps, cleansed and sterilized the canals carefully, and filled the roots at the same sitting, and in only two cases have the patients complained of the teeth being sore, and they were so but a short time, the inflammation subsiding without further treatment.

A case I witnessed with much interest was treated by a young man whom I was trying to help. He was in my office for a few days and never had seen the silver used. The patient, a young woman, had two teeth—the right superior central and lateral incisors—with putrescent pulps. The conditions appeared to be exactly the same. The canal of the central was cleansed in the usual manner, an antiseptic dressing on cotton placed in the root, and the cavity filled with a temporary stopping. The canal of the lateral was also cleansed, and at my suggestion was filled with the silver foil, it first being dipped in an antiseptic, and the cavity in the crown filled permanently. This was all done at the same time. An abscess developed at the roots of the central, which I opened on the third day; but the lateral has not given the slightest trouble up to this time, it being now, if I remember correctly, about six weeks.

For these cases a sufficient quantity is torn from the rope and rolled into a small point, and only such portion used at a time as the operator is able to place in its proper position and condense. I am well aware that any material may have an unmerited record by being used in a run of favorable cases, and in different hands of equal skill the same substance may be used with variable results.

Trusting that silver foil has already found its way into many dental cabinets, that I may be benefitted by the experience of others, and believing that it has germicidal effects when placed in contact with animal tissue, I heartily commend it to your consideration.—*International Journal.*

